# Risk factors for mortality from pneumocystis carinii pneumonia (PCP) in non-HIV patients: a meta-analysis

**DOI:** 10.18632/oncotarget.19927

**Published:** 2017-08-04

**Authors:** Yao Liu, Lili Su, Shu-Juan Jiang, Hui Qu

**Affiliations:** ^1^ Department of Respiratory Medicine, Shandong Provincial Hospital Affiliated to Shandong University, Jinan, Shandong, China; ^2^ Department of General Surgery, Shandong University Qilu Hospital, Jinan, Shandong, China

**Keywords:** non-HIV, pneumocystis pneumonia, risk factor, mortality, meta-analysis

## Abstract

The number of patients with non-human immunodeficiency virus (HIV) related pneumocystis carinii pneumonia (PCP) is increasing with widespread immunosuppressive treatment. We performed a meta-analysis to describe the clinical characteristics and factors associated with outcomes of PCP in HIV-negative patients. A total of 13 studies including 867 patients with non-HIV related PCP was included. The overall mortality for non-HIV patients with PCP was 30.6%. The most common underlying disorder for the development of PCP is hematological malignancies (29.1%), followed by autoimmune disease (20.1%), organ or bone marrow transplantation (14.0%), and solid tumors (6.0%). Risk factors associated with increased mortality rate including old age, female sex, longer time from onset of symptoms to diagnosis, respiratory failure, solid tumors, high lactate dehydrogenase, low serum albumin, bacterial, and aspergillus co-infection, etc (*P* < 0.05). Adjunctive corticosteroid and PCP prophylaxis was not shown to improve the outcome of PCP in non-HIV patients (*P* > 0.05). Our findings indicate that mortality in non-HIV patients with PCP is high. Improved knowledge about the prognostic factors may guide the early treatment.

## INTRODUCTION

Pneumocystis carinii pneumonia (PCP) is widely known as an opportunistic infection in patients with acquired immune deficiency syndrome (AIDS). In recent years, the incidence of PCP in AIDS patients is greatly reduced as the introduction of chemoprophylaxis and highly active antiretroviral therapy [1, 2]. However, PCP in human immunodeficiency virus (HIV)-negative patients increased as the number of patients receiving antitumor chemotherapeutic agents, immunosuppressive therapy, and organ transplantation is growing [1]. Moreover, improved diagnostic procedures may also help to increased detection of non- HIV PCP.

In non-HIV infected patients, PCP tends to follow an acute course, and the reported mortality rate range from 19.6 to 52.9% [3–5], which is significantly higher than the mortality for HIV patients with PCP [1, 6, 7]. Several studies have analyzed clinical manifestations of PCP in patients without AIDS [1, 8–10], and others have tried to determine risk factors associated with mortality for PCP in non-HIV patients [11–13]. However, the sample sizes were relatively modest, and the results were inconsistent. This prompts us to conduct a meta-analysis of published articles in order to determine the underlying diseases or conditions associated with PCP and to indentify risk factors associated with mortality in this population.

## RESULTS

### Study selection

**Figure 1:**
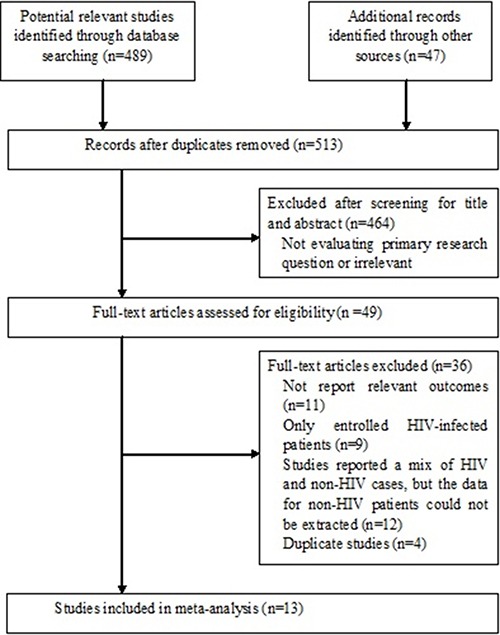
Flow diagram of search strategy and study selection

We initially identified 536 articles, of which 513 remained after removing duplicates (Figure 1). A total of 345 articles were excluded as they were irrelevant to the purpose of our meta-analysis, and a further 119 articles were removed after reviewed the abstracts. Therefore, 49 articles were selected for full-text evaluation and 12 met inclusion criteria. We also searched the reference lists of the 12 articles, and a further one study was identified. A total of 13 articles were included in the review.

### Characteristics of included studies

Baseline characteristics of the 13 studies were shown in Table 1. These studies were from a variety of countries, including 4 from Japan [14–17], 2 from France [9, 18], 2 from Korea [13, 19], and one from each of China [8], Greece [10], US [1], Israel [11], and Taiwan [12]. The sample size of these studies varied from 20 to 173, and the mean age of the patients ranged from 39 to 71 years.

**Table 1:** Characteristics of selected studies

Author	Year	Country	Sample size	Mean age, y	Male, (%)	Diagnosis of PCP	PCP prophylaxis	Steroid use before PCP diagnosis	Antimicrobial agents		Adjunctive steroids	Outcome
TMP-SMX	Others
Hardak [11]	2012	Israel	58	56	30 (52%)	PCR	NA	NA	58 (100%)	0 (0%)	44 (76%)	in-hospital mortality
Kofteridis [10]	2014	Greece	62	65	43 (70%)	Sputum or BAL,	21 (34%)	NA	62 (100%)	0 (0%)	50 (81%)	in-hospital mortality
Mansharamani [1]	2000	US	22	58	22 (67%)	Sputum, BAL, or lung biopsy	0 (0%)	30 (91%)	NA	NA	NA	in-hospital and 1-year mortality
Li [12]	2014	Taiwan	20	50	9 (45%)	PCR	0 (0%)	NA	20 (100%)	0 (0%)	20 (100%)	in-hospital mortality
Kim [13]	2014	Korea	173	56	116 (67%)	PCR, sputum or BAL	6 (3.5%)	128 (74%)	173 (100%)	0 (0%)	152 (88%)	in-hospital mortality
Chen [8]	2015	China	69	39	25 (36%)	PCR, suptum, BAL ,or lung tissue	NA	NA	59(86%)	Clindamycin or primaquine, 10%	58(84%)	in-hospital mortality
Zahar [9]	2002	France	39	52	20(51%)	BAL	5 (13%)	NA	39(100%)	NA	33(85%)	30-day mortality
Asai [14]	2012	Japan	23	71	13(57%)	PCR, sputum or BAL	1 (4.3%)	NA	23(100%)	NA	100%	NA
Matsumura [15]	2011	Japan	82	64	51 (62%)	PCR	3 (4%)	65(79%)	77(94%)	Pentamidine, 6%	60(73%)	30-day mortality.
Tamai [16]	2013	Japan	29	59	14 (48%)	PCR or BAL	1 (3%)	NA	28 (97%)	Pentamidine, 3%	29 (100%)	in-hospital mortality
Roblot [17]	2002	Japan	103	57	61 (59%)	BAL	4 (4%)	57 (55%)	96 (93%)	Atovaquone, 1%; dapsone, 1%	58 (56%)	30-day mortality
Lemiale [18]	2013	France	139	48	79(57%)	BAL	5 (3.6%)	NA	NA	NA	NA	ICU mortality
Ko [19]	2014	Korea	48	53	33(69%)	BAL or lung tissue	14 (29%)	47 (98%)	47 (98%)	Pentamidine, 1%	47 (98%)	in-hospital mortality

PCP, pneumocystis carinii pneumonia; PCR, polymerase chain reaction; NA, not available; BAL, bronchoalveolar lavage; TMP-SMZ trimethoprim sulphamethoxazole. ICU, Intensive Care Unit; TMP-SMX, Trimethoprim/sulfamethoxazole.

There were some variations in the techniques for diagnosis of PCP. The diagnosis of PCP was based on the microbiological identification of pneumocystis carinii in suptum, BAL fluid or lung biopsy specimens in 6 studies [1, 9, 10, 17–19]. The diagnosis of PCP in 4 studies (all after 2011) was made on basis of a positive polymerase chain reaction (PCR) result from a sputum or BAL fluid sample or positive microbiological test [8, 13, 14, 16], and 3 studies only using PCR assay for PCP diagnosis [11, 12, 15] (Table 1).

As first-line treatment, the majority of the patients in the included studies received trimethoprim-sulfamethoxazole (TMP-SMZ) (ranged from 72 to 100%), and other anti-PCP drugs used including pentamidine, atovaquone, dapsone, and clindamycin. The reported proportion of patients received adjunctive corticosteroids range from 56% to100% (Table 1).

Table 2 summarizes the underlying immunosuppressive conditions in patients who suffered from PCP. A total of 252 patients (29.1%) suffered hematological malignancies, 174 (20.1%) had autoimmune disease /chronic inflammatory disease, 52 (6.0%) with solid tumor, and 121 (14.0%) patients receiving organ or bone marrow transplantations. Regarding the type of immunosuppressive treatment, 152 (17.5%) patients were treated with chemotherapy, 188 (21.7%) had received chemotherapy and steroids and 144 (16.6%) had received corticosteroids only.

**Table 2:** The underlying immunosuppressive conditions in HIV-negative patients with PCP

Condition	No of patients (%)	Proportion (%)
*Hematological malignancies, n (%*)	252	29.1
Acute myeloblastic leukaemia	37	4.3
Acute lympoblastic leukaemia	25	2.9
Chronic lympocytic leukaemia	40	4.6
Chronic myeloblastic leukaemia	7	0.81
Lymphoma	98	11.3
Multiple myeloma	19	2.2
Others	26	3.0
*Solid tumors*	52	6.0
Lung cancer	29	3.3
Breast cancer	11	1.3
Stomach cancer	1	0.12
Colon cancer	5	0.58
Cervical cancer	1	0.12
Others	5	0.58
*Autoimmune disease/ chronic inflammatory disease, n (%)*	174	20.1
Systemic lupus erythematosus	51	5.9
Wegener's granulomatosis	1	0.12
Reumatoid arthritis	53	6.1
Sarcoidosis	3	0.35
dermatomyositis	20	2.3
Chronic kidney disease	0	0
Autoimmune hepatitis	2	0.23
Others	43	5.0
*Organ or bone marrow Transplantation*	121	14.0
*Type of immunosuppressive treatment*	484	55.8
Chemotherapy alone	152	17.5
Steroids + chemotherapy	188	21.7
Steroids	144	16.6

The synthesis of the main results for the 23 potential risk factors was showed in Table 3. Among the 23 factors included in this study, 13 factors were significantly associated with increased odds of death from PCP, whereas 2 factors were significantly associated with decreased risk of mortality.

**Table 3:** Synthesis of the main results from this systematic review

Category of risk factor	Risk factors	Comparisons	No of studies included in the meta-analysis	No of patient include in the meta-analysis	Pooled OR/WMD (95% CI)	*P*	*I*^2^(%)
Demographic factors	Age	Age (years) mean ± SD	9	657	6.33 (3.45–9.21)	< 0.0001	0
	Gender	Female vs Male	11	1531	1.43 (1.12–1.83)	0.004	0
	Respiratory failure	Yes vs No	4	203	6.16 (2.57–14.77)	< 0.0001	0
	ICU admission	Yes vs No	2	82	4.85 (1.28–18.38)	0.02	0
	Smoker	Yes vs No	3	1066	0.97 (0.69–1.38)	0.88	0
	Time from onset of symptoms to diagnosis	Time (days), mean ± SD	4	16	3.53 (0.73–6.33)	0.01	0
Underlying diseases	Haematological malignancy	Yes vs No	7	541	0.64 (0.44–0.92)	0.02	32
	Solid tumor	Yes vs No	6	548	2.66 (1.72–4.13)	< 0.0001	0
	Autoimmune disease	Yes vs No	6	411	1.07 (0.67–1.69)	0.78	67
	Organ transplantations	Yes vs No	5	369	0.38 (0.20–0.74)	0.004	39
Symptom	Fever	Yes vs No	4	1152	0.91 (0.63–1.33)	0.64	0
	Dyspnea	Yes vs No	3	1070	1.50 (1.10–2.04)	0.01	0
	Cough	Yes vs No	2	235	0.68 (0.39–1.18)	0.17	0
Laboratory findings	LDH	LDH (U/L) mean ± SD	9	754	151 (90–212)	< 0.0001	7
	Neutropenia (< 1500/mL)	Yes vs No	2	120	1.43 (0.56–3.66)	0.45	0
	Albumin	Albumin g/L, mean ± SD	6	463	-0.39 (-0.49 - -0.28)	< 0.0001	52
Presence of co-infection	Bacterium	Yes vs No	7	574	2.17 (1.34–3.51)	0.002	25
	Cytomegalovirus	Yes vs No	4	199	2.33 (1.15–4.71)	0.02	40
	Aspergillus	Yes vs No	2	151	10.45 (2.79–40.45)	0.0007	0
Treatment	PCP prophylaxis	Yes vs No	5	1196	0.97 (0.69–1.34)	0.83	0
	Adjunctive steroids	Yes vs No	6	445	1.15 (0.72–1.82)	0.55	0
	Previous Corticosteroid	Yes vs No	3	254	1.05 (0.54–2.03)	0.88	0
	Mechanical ventilation	Yes vs No	7	558	23.46 (14.02–39.28)	< 0.0001	35

OR, Odds Ratio; WMD, weighted mean difference; ICU, Intensive Care Unit; PCP, pneumocystis carinii pneumonia; LDH, lactate dehydrogenase.

### Demographic variables

Among demographic variables at admission, five variables were significantly associated with increased risk of mortality: old age (MD 6.33 y, 95% CI 3.45 to 9.21 y, *P* < 0.001); female sex (OR 1.43, 95% CI 1.12 to 1.83, *P* = 0.004); respiratory failure (OR 6.16, 95% CI 2.57 to 14.77, *P* < 0.001); ICU admission (OR 4.85, 95% CI 1.28 to 18.38, *P* = 0.02); and time from onset of symptoms to diagnosis (MD 3.53 d, 95% CI 0.73 to 6.33 days, *P* = 0.01) (Table 3).

### Underlying diseases

Solid tumor was found to be associated with increased risk of death from PCP (OR 2.66, 95% CI 1.72 to 4.13, *P* < 0.001). Haematological malignancy (OR 0.64, 95% CI 0.44 to 0.92, *P* = 0.02) and organ transplantations (OR 0.38, 95% CI 0.20 to 0.74, *P* = 0.004) were associated with significant lower odds of mortality from PCP. Autoimmune disease did not appear to increase the risk of mortality (OR 1.07, 95% CI 0.67 to 1.69, *P* = 0.78) (Table 3).

### Symptoms

The common clinical symptoms reported in patients with PCP were fever, dyspnea and cough. Dyspnea was associated with an increased risk of death (OR 1.50, 95% CI 1.10 to 2.04, *P* = 0.01). Fever (OR 0.91, 95% CI 0.63 to 1.33, *P* = 0.64) or cough (OR 0.68, 95% CI 0.39 to 1.18, *P* = 0.17) was non-significantly associated with mortality risk (Table 3).

### Laboratory variables

Among laboratory variables at admission, a higher lactate dehydrogenase (LDH) level (MD 151 U/L, 95% CI 90 to 212 U/L, *P* < 0.001), and lower serum albumin level (MD −0.39, 95% CI −0.49 to −0.28, *P* < 0.001) were significantly associated with mortality. Neutropenia (< 1500/mL) did not significantly influence mortality (OR 1.43, 95% CI 0.56 to 3.66, *P* < 0.001) (Table 3).

### Presence of co-infection

Among co-infection recorded during hospitalization, a concomitant infection of bacteria (OR 2.17, 95% CI 1.34 to 3.51, *P* = 0.002), cytomegalovirus (OR 2.33, 95% CI 1.15 to 4.71 *P* = 0.02), or aspergillus infection (OR 10.45, 95% CI 2.79 to 40.75, *P* = 0.0007) was each significantly associated with mortality (Table 3).

### Treatment variables

**Figure 2:**
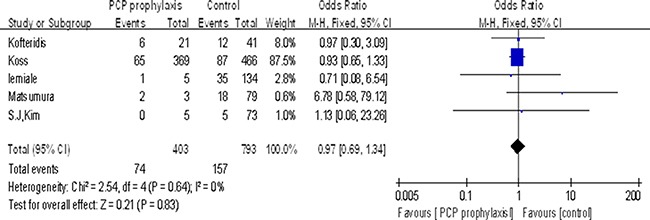
Association between PCP prophylaxis and mortality

Five studies reported the effect of PCP prophylaxis on mortality. A total of 403 of the 1196 patients (34%) had received TMP-SMZ prophylaxis for PCP before the disease onset. Findings from the pooled analysis suggested there was no significant association between PCP prophylaxis and death (OR 0.97, 95% CI 0.69 to 1.34, *P* = 0.83) (Figure 2).

**Figure 3:**
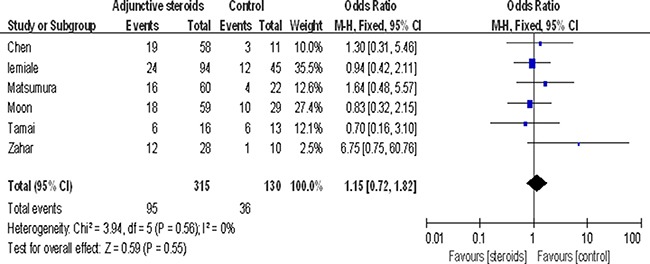
Association between adjuvant steroids and mortality

Six studies evaluated the effect of adjuvant steroids for mortality (*n* = 445). Adjuvant steroids were given to 315 (71%) patients. Findings from this analysis suggested adjuvant steroid therapy did not significantly influence mortality (OR 1.15, 95% CI 0.72 to 1.82, *P* = 0.55) (Figure 3).

The risk of mortality was also not influenced by the steroid treatment before admission (OR 1.05, 95% CI 0.54 to 2.03, *P* = 0.88). Use of immunosuppressive agents before the diagnosis of PCP was associated with decreased odds of death (OR 0.29, 95% CI 0.12 to 0.73, *P* = 0.009). Hematologic/oncology patients receiving chemotherapy (OR 4.24, 95% CI 1.81 to 9.92, *P* = 0.0009), and a need for mechanical ventilation (OR 23.46, 95% CI 14.02 to 39.28, *P* < 0.0001) were each associated with significantly increased risk of mortality (Table 3).

### Risk of bias, sensitivity and subgroup analyses

Supplementary Table 1 reported the risks of bias for the included studies: 4 of the 13 studies were considered to be at high risk of bias, and lack of adjustment for confounding (statistical analysis bias) was the most frequent risk of bias.

In sensitivity analyses, we removed the 4 studies with high risk of bias. Overall, for most of the factors significantly associated with mortality, no substantial change was observed after sensitivity analyses. After removing the studies with high risk of bias, only time from onset of symptoms to diagnosis lost statistical significance (MD 3.50 d, 95% CI −5.77 to 12.77 days, *P* = 0.46).

In the subgroup analyses, we compared the associations between risk factors and death in subsets of studies grouped by different techniques for PCP diagnosis (PCR method or cytologic identification of pneumocystis carinii) (Supplementary Table 2). The results were not substantially changed by subgroup analysis. Gender, haematological malignancy, lower albumin level and bacterial co-infection were not significantly affect mortality in the subgroup of studies using cytologic method for diagnosis (*P* > 0.05) (Supplementary Table 2).

### Publication bias

Funnel plots and Egger's tests did not suggest a publication bias for the majority of the risk factors examined with the exception of autoimmune disease, albumin, or chemotherapy.

## DISCUSSION

Despite the number of patients with HIV-negative PCP is increasing with widespread chemotherapy and immunosuppressive treatment, the clinical characteristics and the risk factors associated with mortality of non-HIV PCP have not been well defined. Our meta-analysis comprehensively reviewed 13 studies involving 867 patients with non-HIV related PCP which described the clinical courses, and prognosis factors in these patients. The results showed many factors including demographic, underlying diseases, symptomatic, laboratory, presence of co-infection and treatment variables were associated with increased odds for mortality. These clinical factors are easily to be monitored and measured in the primary care setting. To our knowledge, this study is the first systematic review on this topic.

All patients in our meta-analysis had an immunosuppressive condition for PCP. The pooled analysis showed the most common underlying disorder for the development of PCP is hematological malignancies (29.1%) with leukemia (12.6%) or lymphomas (11.3%), followed by autoimmune disease (20.1%) (particularly systemic lupus erythematosus and reumatoid arthritis), organ or bone marrow transplantation (14.0%), and solid tumors (6.0%). The variety of diseases observed suggested that PCP should be suspected in many immunocompromised patients and that special stainings or PCR assays for the detection of pneumocystis DNA should be considerated for diagnosis. The majority of the study population had a history of receiving immunosuppressive treatment (eg, steroids or steroids in combination with anti-malignancy chemotherapy). However, it should be note that 18% of the patients in this study were receiving only chemotherapy, when PCP occurred. This finding is in accordance with previous studies examining PCP in cancer patients and may indicate that chemotherapy or even malignancy only can increase the PCP risk [20, 21].

Our results are consistent with previous data for HIV-positive patients that mortality was influenced by respiratory failure, high lactate dehydrogenase, low serum albumin, concomitant bacterial infection, and a need for mechanical ventilation [4, 6, 7, 22–24]. Many other findings in this meta-analysis were novel and deserve further discussion.

Among demographic variables, old age, female sex, longer time from onset of symptoms to diagnosis was associated with significant increased risk of mortality. Previous study showed the duration between admission and the initialization of PCP treatment was longer in the non-HIV infected group than HIV-infected group [12]. Such a delay in treatment is likely to be related to the delay in the diagnosis of PCP, for which the clinical manifestations and radiologic abnormalities are nonspecific. Improved diagnostic procedures aiming to reduce the delay between symptoms onset and the diagnosis in HIV-negative patients would be important.

Solid tumor was one of the comorbidities associated with increased risk of death from PCP. This result is consistent with a recent study reporting prognosis seems to be worse in patients with solid tumors [9]. In this study, we also found that co-infection, most notably with aspergillosis, was associated with higher mortality. Non-HIV immunocompromised patients are more vulnerable to aspergillosis infection [25], it is possibly because both PCP and aspergillosis develop with the use of immunosuppressive agents [17, 25].

Despite the presence of multiple major risk factors, PCP prophylaxis was not generally implemented for immunocompromised non-HIV patients. Our review showed that 10.4% (34/327) of HIV-negative patients with PCP were on PCP prophylaxis before the onset of disease. Although no significant differences were observed in mortality in the present study between patients with and without TMP-SMX prophylaxis, a previous systematic meta-analysis including 1245 patients who had undergone bone marrow or solid organ transplant or with hematologic cancer showed that there was a 91% reduction in the occurrence of PCP in non-HIV patients who received prophylaxis [26]. In an observational study, it was suggested that PCP prophylaxis should be given to all solid organ transplant recipients for at least 1 year [27]. Indeed, PCP prophylaxis is recommended for all organ transplant recipients for at least 6–12 months post transplantation [28]. However, limited data are available for the efficiency of PCP prophylaxis in solid tumors and immunosuppressed patients with rheumatic diseases. It has been reported that, PCP was most likely to occur during the reduction or withdrawal of chemotherapy or even during clinical remission [3]. Other study showed patients with uncontrolled tumor growth were candidates to develop PCP [9]. Further well-designed prospective research should be undertaken to define the type of solid tumor patients who may benefit from PCP prophylaxis.

For HIV-infected patients with PCP, many studies showed that adjunctive corticosteroid treatment prevented early deterioration [29], and reduced the occurance of respiratory failure and mortality rate [30, 31]. Based on these data, adjunctive corticosteroid is recommended to AIDS patients with PCP, if PaO_2_ is ≤ 70 mmHg or AaDO_2_ is ≥ 35 mmHg [32]. However, there is no proof that HIV-negative patients might benefit from adjunctive corticosteroid. Two previous studies analyzed the effect of corticosteroid on non-HIV patients with severe PCP (defined by an PaO_2_ < 70 mm Hg), which results suggested adjunctive steroid therapy possibly accelerated recovery but failed to reduce the rates of endotracheal mechanical ventilation and in-hospital mortality [33, 34].

Similarly, our pooled analyses of 6 articles support the results of previous studies that adjunctive corticosteroid treatment may not improve the prognosis of PCP in non-HIV patients [33, 34]. However, it should be noted that the non-HIV-immunocompromised patients were not a homogeneous group, and most of them were on corticosteroid treatment at the time they developed PCP. The data on steroid treatment, comorbidity and disease severity can be confounded. More research is needed in this area to better understand how adjunctive corticosteroid influencing the mortality of HIV-negative PCP patients in different settings.

In our study, the pooled overall mortality for non-HIV patients with PCP was 30.6%, which was significantly higher than previously reported mortality rate in HIV-positive patients. The present data confirms the results of previous studies that HIV-negative patients with PCP have different prognosis from the HIV-positive ones [7, 13, 24]. There are several possible explanations for the poorer outcomes in non-HIV PCP. HIV-negative patients were older and had more underlying cardiopulmonary disease than HIV-positive patients. The duration of symptoms onset to the beginning of PCP treatment was much longer in HIV-negative patients. Moreover, AIDS patients with PCP were benefited from adjunctive steroid therapy, but there is no proof that adjunctive corticosteroid is beneficial to HIV-negative patients. This suggests that non-HIV PCP may not benefited from the advances in the management of PCP.

Although we believed that the current study provided useful information, some potential limitations should be noted. Firstly, many studies differed in their study population, era, methods of diagnosis of PCP, and follow-up. The method for PCP diagnosis is a major cause of heterogeneity. When compared with traditional cytological stains, PCR detection of pneumocystis carinii DNA provides greater sensitivity, but carries the risk of false-positive diagnosis of PCP in patients who are not infected, but colonized, with pneumocystis carinii [35]. Colonization with pneumocystis carinii refer to when a patient did not have specific symptoms or history of PCP, and showed a positive nested-PCR result indicating presence of pneumocystis carinii DNA in his or her respiratory secretions [35]. Pneumocystis carinii colonization often occurs in patients with underlying pulmonary diseases and mild immunosuppression [36]. The colonized patients differed from the PCP infected patients, since they presented with lower quantitative PCR assay value [37]. Moreover, recent data demonstrated that the (1,3) ß-D-glucan (BDG) level in bronchoalveolar lavage (BAL) sample from the PCP patients was significantly higher than the colonized patients [38]. For ambiguous PCR results, BDG could be used as a preliminary test for patients with suspected PCP, especially in patients with slightly positive PCR results [39]. Last, most of the studies presented only unadjusted estimates, it was not possible to stratify or adjust for potential confounders in this meta-analysis, which restricted us to obtain more comprehensive results and do further detailed analysis.

In summary, our study suggests all immunocompromised non-HIV patients with symptoms of pulmonary infection should be carefully evaluated for PCP, and the mortality rate is still high in non-HIV infected patients with PCP. Risk factors associated with poor prognosis including old age, female sex, longer time from onset of symptoms to diagnosis, respiratory failure, solid tumors, high LDH, low serum albumin, concomitant infection, etc. The identification of high risk non-HIV patients with PCP has great clinical relevance with respect to patients counsel, and guides the early treatment. Limited data are available for the efficiency of PCP prophylaxis in solid tumors and patients with rheumatic diseases. Further studies evaluate the role of PCP prophylaxis and adjunctive corticosteroid in immunocompromised patients PCP with should be advocated.

## MATERIALS AND METHODS

This systematic review and meta-analysis was conducted following the PRISMA statement [40].

### Search strategy

We conducted a systematic search of PubMed, Embase, and Scopus Database for articles published until Dec 2016. The text keywords used in searching included: “Pneumocystis Infections ” or “pneumocystis” or “carinii” or “jiroveci” or “PCP ” or “PJP ” combined with “outcome ” or “mortality”’. No language restriction was applied. From the title or abstract, the literature search was reviewed by 2 authors (YL and SJJ) independently to identify potentially relevant studies for full text review. The “related articles” function was used to broaden the search. Moreover, a manual search of references from related articles was performed to identify additional relevant studies.

### Study selection

Original articles were considered to be eligible if they investigated the predisposing factors, clinical characteristics and reported mortality data of PCP in HIV-negative patients. Studies restricted to HIV-infected participants or children patients were excluded. Letters, meeting proceedings, and abstracts were also excluded. Studies which reported a mix of HIV and non-HIV infected cases were also included if sufficient data were provided to extract non-HIV infected patients meeting the inclusion criteria. After obtained the full text of candidate studies, the two authors (YL and SJJ) independently assessed eligibility. Disagreements were resolved by reviewing corresponding articles.

### Data extraction

Two authors (YL and SJJ) extracted data from included studies. Differences between the two authors were solved by discussion between the two authors and consensus with a third author (LLS). We extracted the following information: first author's last name, publication year, study design, study population, sample size, methods for PCP diagnosis, potential factors associated with mortality including demographical details, underlying diseases, clinical characteristics, laboratory results, treatment variables and et al.

### Statistical analyses

The statistical analyses were performed with Stata (version 12.0; Stata Corporation, College Station, TX, USA) and Revman (version 5.2; Cochrane Collaboration, Oxford, United Kingdom). When meta-analysis was possible, for each risk factor we generated a pooled OR or mean difference (MD) using the inverse-variance weighting method. If both of the univariate and multivariate regression results were reported, we used estimates from the multivariate regression model. As we expected high heterogeneity in the subjects, the diagnosis of PCP, the definition of risk factors, we selected a priori the DerSimonian and Laird random effect model. We tested for heterogeneity with the Cochrane *Q* test and measured degree of heterogeneity across studies using the I-squared (*I*^2^) statistic. The degree of heterogeneity between studies was assessed by I^2^ statistic with its 95% confidence intervals. Heterogeneity was considered low for I^2^ values between 25%–50%, moderate for 50%–75%, and high for 75% [41].

The method for PCP diagnosis may be an important source of heterogeneity. In order to analyze the heterogeneity, subgroup analyses were performed by comparing summary results obtained from subsets of studies grouped by different diagnosis methods (PCR method or cytologic identification of pneumocystis organisms). If at least two studies for each subgroup were available, subgroup analyses were performed. We also performed sensitivity analyses were to examine the effect of the risk factors after removing the studies with high risk of bias. We investigated the presence of publication bias by means of funnel plots and Egger's test.

## SUPPLEMENTARY MATERIALS TABLES


