# Supplementary material for: Risk factors for mortality from pneumocystis carinii pneumonia (PCP) in non-HIV patients: a meta-analysis
Source: Oncotarget. 2017 Aug 4;8(35):59729–39. doi: 10.18632/oncotarget.19927 (PMC5601772; doi:10.18632/oncotarget.19927)
Supplement: Supplementary file 1 [file oncotarget-08-59729-s001.pdf]

Risk factors for mortality from pneumocystis carinii pneumonia (PCP) in non-HIV patients: a meta-analysis

SUPPLEMENTARY MATERIALS

Supplementary Table 1: Appraisal of the risk of bias of the included studies

	Study participation	Study Attrition	Risk factors measurement	Outcome measurement	Study confounding	Statistical analysis	Overall risk of bias
Hardak, 2012							high
Kofteridis, 2014							
Mansharamani, 2000							high
Li, 2014							high
Kim, 2014							
Chen, 2015							
Zahar, 2002							
Asai, 2012							
Matsumura, 2011							high
Tamai, 2013							
Roblot, 2001							
Lemiale, 2013							
Ko, 2014							

Supplementary Table 2: Subgroup analysis according to the method for PCP diagnosis

		Diagnosis of PCP					
		PCR method			Cytologic identification of Pneumocystis organisms		
Risk factors	Comparisons	No of studies	Pooled OR/WMD (95% CI)	I^2 (%)	No of studies	Pooled OR/WMD (95% CI)	I^2 (%)
Age	Age (years) mean ± SD	6	7.81 (4.50–11.13)	0	3	1.89 (−3.86–7.64)	0
Gender	Male vs Female	5	0.54 (0.33–0.89)	29	6	0.76 (0.57–1.01)	0
Respiratory failure	Yes vs No	2	5.89 (1.46–23.80)	0	2	6.41 (2.16–19.00)	56
Haematological malignancy	Yes vs No	4	0.62 (0.36–1.05)	59	3	0.66 (0.39–1.10)	0
Solid tumor	Yes vs No	3	1.91 (1.00–3.64)	0	3	3.54 (1.93–6.51)	35
Autoimmune disease	Yes vs No	4	1.22 (0.69–2.16)	75	2	0.82 (0.36–1.82)	59
Organ transplantations	Yes vs No	3	0.46 (0.21–1.09)	61	2	0.29 (0.10–0.81)	0
LDH	LDH (U/L) mean ± SD	3	137 (71–204)	18	6	226 (70–381)	0
Albumin	Albumin g/L, mean ± SD	4	−0.44 (−0.55−0.33)	0	2	0.09(−0.25- 0.42)	0
Bacterium	Yes vs No	4	3.29 (1.66–6.52)	0	3	1.41(0.70–2.84)	2
PCP prophylaxis	Yes vs No	2	3.21 (0.59–17. 35)	0	3	0.93 (0.66–1.30)	0
Adjunctive steroids	Yes vs No	3	1.21 (0.56–2.63)	0	3	1.12(0.63–1.98)	36
Previous Corticosteroid	Yes vs No	2	1.73 (0.91–3.27)	0	4	1.78 (0.70–4.54)	0
Mechanical ventilation	Yes vs No	3	31.01 (13.62–70.61)	75	4	18.43 (9.47–35.89)	0

PCP, pneumocystis carinii pneumonia; PCR, polymerase chain reaction; OR, Odds Ratio; WMD, weighted mean difference; LDH, lactate dehydrogenase.
